# Design, Modeling, and Experimental Validation of an Active Microcatheter Driven by Shape Memory Effects

**DOI:** 10.3390/mi15050603

**Published:** 2024-04-30

**Authors:** Chengyang Li, Xu Zhang, Zhongjing Ren, Jingkai Wang, Shouyu Sun, Jian Fu, Yang Xu, Wu Duan

**Affiliations:** 1 School of Mechanical Engineering, Shandong University, Jinan 250061, China; lcy09172022@163.com (C.L.); 15685752640@163.com (X.Z.); chuans058@126.com (J.W.); sunshouyu@andemed.com (S.S.); 2 Shenzhen Research Institute of Shandong University, Shenzhen 518057, China; 3 School of Electrical and Information Engineering, Lanzhou University of Technology, Lanzhou 730050, China; 4 Zhejiang Julihuang Sawing Machine Group Co., Ltd., Lishui 321404, China; 5 Shandong Ande Healthcare Apparatus Co., Ltd., Zibo 255086, China; fujian@andemed.com; 6 Department of Mechanical Engineering, Stevens Institute of Technology, Hoboken, NJ 07030, USA; yxu55@stevens.edu; 7 Department of Endocrinology, Qilu Hospital of Shandong University, Jinan 250012, China

**Keywords:** microcatheter, shape memory effect (SME), active navigation, vascular intervention

## Abstract

Microcatheters capable of active guidance have been proven to be effective and efficient solutions to interventional surgeries for cardiovascular and cerebrovascular diseases. Herein, a novel microcatheter made of two biocompatible materials, shape memory alloy (SMA) and polyethylene (PE), is proposed. It consists of a reconfigurable distal actuator and a separate polyethylene catheter. The distal actuator is created via embedding U-shape SMA wires into the PE base, and its reconfigurability is mainly dominated by the shape memory effect (SME) of SMA wires, as well as the effect of thermal mismatch between the SMA and PE base. A mathematical model was established to predict the distal actuator’s deformation, and the analytical solutions show great agreement with the finite element results. Structural optimization of such microcatheters was carried out using the verified analytical model, followed by fabrication of some typical prototypes. Experimental testing of their mechanical behaviors demonstrates the feasibility of the structural designs, and the reliability and accuracy of the mathematical model. The active microcatheter, together with the prediction model, will lay a solid foundation for rapid development and optimization of active navigation strategies for vascular interventions.

## 1. Introduction

The past few decades have witnessed cardiovascular and cerebrovascular diseases caused by thrombus formation becoming a major threat to human life [1,2]. Continuous robots with active guidance functions have shown great potential to revolutionize the field of interventional surgery, since they can change their shape and bending stiffness to adapt to complex surroundings [3,4]. However, most of the existing continuous robots have proven unavailable for interventional catheters functioning in blood vessels that possess complicated vascular lumens with diameters at the millimeter scale [5,6,7]. Researchers have proposed various active microcatheters with adaptive driving methods to address this issue [8,9]. Leber A. et al. [10] developed a multi-material fiber driven by a tendon-driven mechanism and fabricated using a thermal drawing technique. This catheter could achieve complex motion due to its unique structures and control strategy. Gopesh T. et al. [11] introduced a fabrication process for a hydraulically driven soft polymer tip that can be attached to the distal end of a microcatheter, providing active navigation ability. However, the manufacturing difficulty and stiffness of the tip based solely on soft materials need to be carefully considered. Liu, X. et al. [12] proposed a magnetic soft microfiberbot with high steerability and multimodal shape reconfigurability, which is fabricated by thermal drawing of a magnetic soft composite into microfibers. Kim, Y. et al. [13,14] presented a magnetically driven submillimeter linear continuum robot made of ferromagnetic soft material. However, the manipulation of this magnetic robot requires not only expensive equipment to provide the required magnetic field, but also other ancillary equipment, such as robotic arms, all of which hinder its widespread use [15,16].

Besides the driving methods mentioned above, researchers have also explored the approach to designing active microcatheters using shape memory effects [17,18]. In this study, a combination of two different materials is proposed for the design of active microcatheters. Shape memory alloys are widely used in different situations due to their remarkable shape memory effects, as well as their biocompatibility [19,20,21]. Once being heated beyond its austenite finish temperature, proper annealed SMA would regain its memorized shape at a much higher annealing temperature, eliminating its macroscopic deformation occurring in the martensitic phase. Shape memory alloys with large strains up to 8% have been commonly used in actuation elements, providing a significant driving force during their stroke course [22,23,24]. As such, SMA wires with submillimeter diameters and appropriate annealing for SME actuation greatly appeal to researchers working on developing actively controllable microcatheters for blood vessels with inner diameters of several millimeters [25,26,27]. Multiple polymers, such as polydimethylsiloxane (PDMS), hydrogel, silicone rubber, and polyethylene (PE)., have been prevalently used as the base materials of microcatheters [28,29,30,31]. As a biocompatible material, polyethylene is already used in a variety of medical devices, and in addition, it has a higher Young’s modulus than silicone rubber, which can achieve a better stiffness match with SMA. However, the theoretical modeling of such active microcatheters remains unsolved. It is worth pointing out that geometries and dimensions of such excellent thermoplastic resin polymers should be taken care of, so as to ensure the efficient reconfiguration under the combined effects of thermal mismatch and SME. Therefore, it is meaningful to devise a strategy for rapid prediction and structural optimization of these microcatheters’ mechanical performance.

Our previous research on this topic has successfully demonstrated that the morphing efficiency of microstructures consisting of two materials with different coefficients of thermal expansion can be significantly improved via the introduction of shape memory effects [32,33,34,35,36,37,38]. In this work, we focus on the structural design and mathematical modeling of a novel microcatheter for vascular interventions. The analytical solution to such a mathematical model verified by the finite element method offers an efficient tool for rapid evaluation and optimization of the proposed microcatheters. This paper is organized as follows. Firstly, the structural design and working mechanism of the microcatheter are introduced in Section 2. Then, in Section 3, the mathematical model of its mechanical performance under thermal actuation is established, which is compared to the finite element models built using the finite element analysis software ABAQUS 2021. After that, fabrication and experimental testing of the proposed microcatheter are completed in Section 4. Finally, Section 5 concludes this work. 

## 2. Structural Design and Working Mechanism

**Figure 1:**
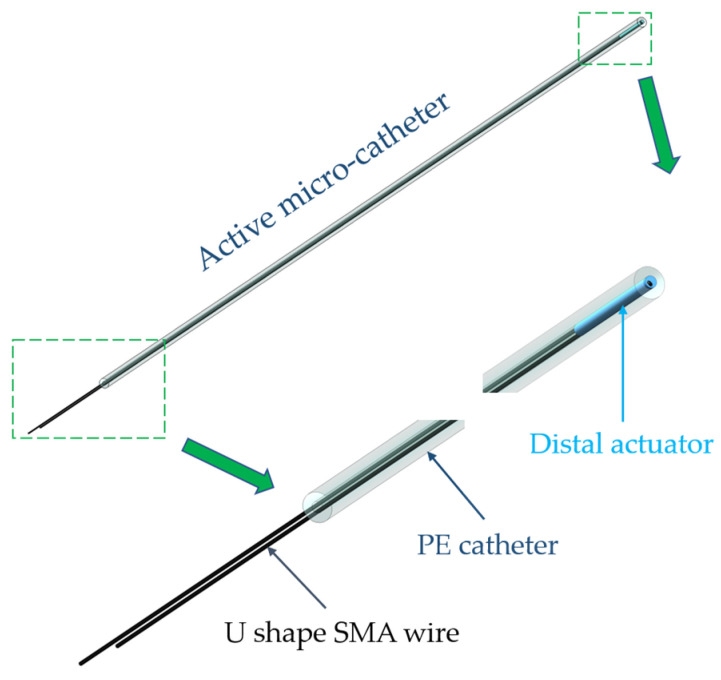
Conceptual design of the presented active microcatheter.

**Figure 2:**
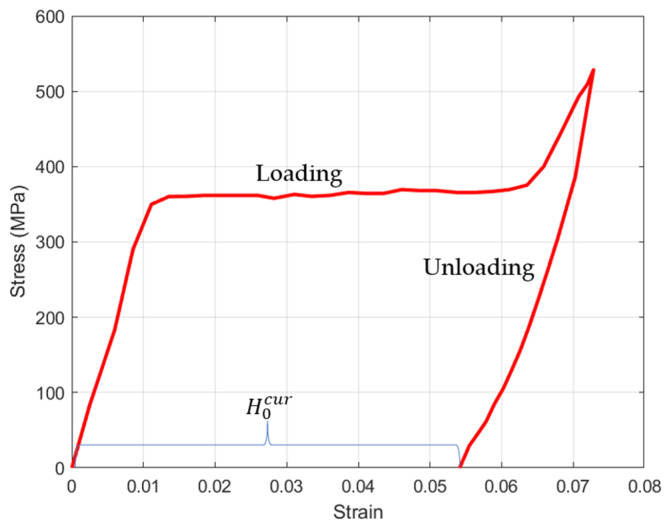
Stress–strain behavior of the SMA during the loading and unloading process.

In this work, PE and NiTi SMA were employed to fabricate the distal actuator of the proposed active microcatheter, which consisted of a distal actuator and a PE catheter, as shown in Figure 1. Young’s modulus of the SMA wire with a diameter of 0.1 mm was characterized by a tensile tester. As a result, the stress–strain behavior of the NiTi SMA wire (in twined martensitic phase) is presented in Figure 2. Then, Young’s modulus of the NiTi SMA, as well as its maximum residual strain, can be acquired. NiTi SMA wires were used for developing the microcatheter. Initially, the SMA wire in the detwinned phase, which had a residual strain of approximately 5%, was partially processed by tensile stress at room temperature (25 °C), providing the driving force for active navigation of the microcatheter. As such, the distal actuator was designed as a PE body with the detwinned U-shaped SMA wire embedded in a biased manner.

Young’s modulus of the SMA in the martensite phase is smaller than that in the austenite phase, and Young’s moduli of the SMA in both phases are higher than that of PE. The working mechanism of the proposed active microcatheter is explained as follows. When the SMA wire is heated to over the austenite finish temperature via Joule heating, the distal actuator undergoes efficient deformation due to the significant contraction of the SMA wire arising from its shape memory effect and the partial release of residual stress stored in the SMA wire. Meanwhile, the bending of the PE base under the impact of the SMA wire retains a certain amount of potential energy at the distal actuator. When the temperature of the SMA wire drops below the martensite finish temperature, the potential energy stored in the distal actuator is released, partially restoring its original configuration.

**Figure 3:**
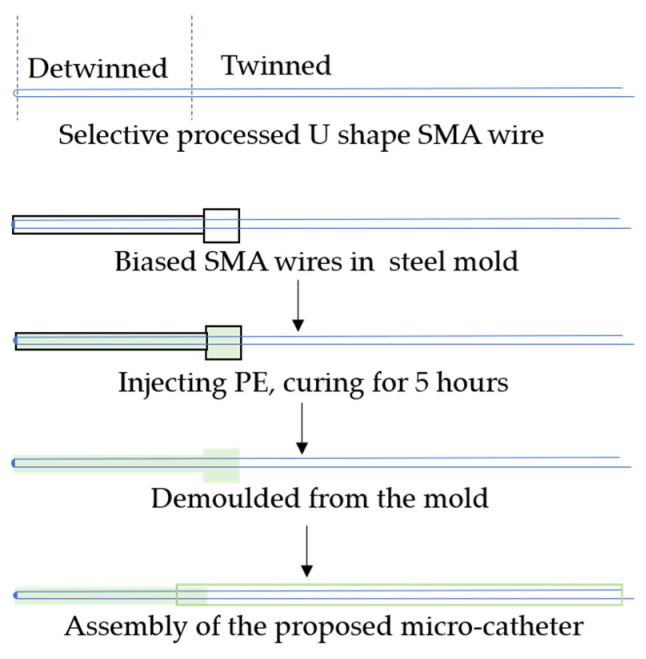
The manufacturing process diagram of the active microcatheter.

The typical manufacturing process of the proposed microcatheter in this paper is illustrated in Figure 3. First, the distal part of the SMA wire undergoes a tensile stress treatment to complete the detwinning process, while the remaining part is maintained in the twinning martensite phase. Then, the SMA wire is biasedly placed in a linear steel mold with an inner diameter of less than 1 mm. Next, liquid PE is injected into the mold and left to cure for four hours. Afterward, the cured PE base, along with the detwinned SMA wire, can be pulled out of the mold using a tensile testing device. Finally, by assembling the distal actuator with a separate PE catheter, a prototype of the designed active microcatheter can be obtained.

## 3. Mathematical Model and Simulation

In this section, a mathematical model is proposed to evaluate the degree of curvature of the active microcatheter, and the bending process of the active microcatheter is simulated by the finite element method. Finally, the results obtained by the two methods are compared to prove that the mathematical model presented in this paper is effective.

### 3.1. Mathematical Modeling of the Microcatheter

Active navigation of the microcatheter mainly depends on the deflection of the distal actuator. To predict and evaluate the deformation performance of the actuator, an analytical solution to the static model of the bending capacity of the microcatheter is desired. It is worth noting that the bending operation is driven by the maximum contraction strain resulting from the reverse phase transformation of the SMA wires from martensite to austenite. Therefore, we can acquire the negative thermal strain due to the maximum residual strain ($H_{0}^{cur}$) of the detwinned SMA wires:

(1) $$\varepsilon_{0}=-\frac{H_{0}^{cur}}{1+H_{0}^{cur}}$$

where the negative sign in Equation (1) implies the compressive strain in SMA wires arising from their shape memory effects. It is evident that the magnitude of the contraction strain is dominated by the residual strain induced by the loading on the SMA wire during the detwinning process.

**Figure 4:**
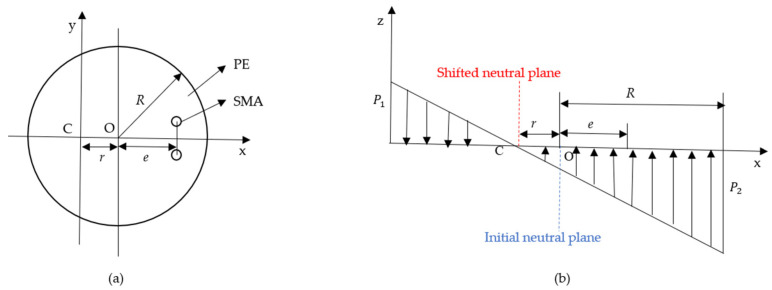
Stress analysis of the cross-section of the distal actuator: (**a**) schematic diagram of the cross-section; (**b**) schematic diagram of stress distribution.

The compressive stress induced by the austenitic phase transition in biased SMA wires can contribute to the significant bending deflection of the PE base. Note that the neutral plane of the cross-section of the tip actuator shifts away from the neutral plane of the PE base. Herein, we propose a prediction method for determining the neutral plane’s shift. As seen in Figure 4a, structural parameters of *R*, *e*, and *r* represent the radius of the PE cross-section, the vertical distance between the center of the cross-section and the line of the SMA wire’s center, and the offset distance of the neutral plane, respectively. Moreover, the analysis of the stress distribution across the cross-section of the distal actuator is schematically depicted in Figure 4b. The maximum tensile stress and compressive stress at the edge of the cross section are denoted $P_{1}$ and $P_{2}$, respectively. The symbols r and $k_{p}$ represent the offset of the neutral plane and the stress change rate of the cross-section with the distance from the center of the circle, respectively.

As such, the stresses $P_{1}$ and $P_{2}$ can be expressed as:

(2) $$P_{1}=k_{p}\left(R-r\right)$$

(3) $$P_{2}=k_{p}\left(R+r\right)\;$$

Once the temperature of the SMA wire is heated above the austenitic finish temperature, the PE-based tip actuator would be driven by the internal stress and moment, and reach an equilibrium state. The formula for description of the deformation can be obtained as:

(4) $$k_{p}\int_{r-R}^{r+R}2x\sqrt{R^{2}-{\left(x-r\right)}^{2}}dx=F$$

(5) $$k_{p}\int_{r-R}^{r+R}2x^{2}\sqrt{R^{2}-{\left(x-r\right)}^{2}}dx=F(e+r)$$

Note that the sign of *F* in Equations (4) and (5) implies the force resulting from the contraction strain of the detwinning SMA wires. Equations (4) and (5) can be solved as:

(6) $$F=\pi R^{2}k_{p}r$$

(7) $$F\left(e+r\right)=\pi R^{2}k_{p}(r^{2}+\frac{R^{2}}{4})\;\;$$

By combining Equations (6) and (7), we obtain the relationship between the parameters *r*, *e*, and *R*, that is:

(8) $$r=\frac{R^{2}}{4e}$$

According to Figure 4, it is obvious that the sum of *r* and *e* represents the distance between the center of SMA wires and the shifted neutral plane. In this work, we propose a method to estimate the bending curvature of the distal actuator based on the internal stress and moment in SMA wires. By employing the Euler–Bernoulli beam theory, the curvature of the distal actuator can be written as:

(9) $$\kappa =\frac{M}{EI}$$

where *M* is the internal moment applied on the PE base arising from the contraction of the SMA, and *E* and *I* represent Young’s modulus of PE and the moment of inertia of the PE base’s cross-section, respectively. Once the distal actuator is in bending equilibrium, the moment formed by the tensile force of the SMA wires embedded into the PE base is balanced with the PE’s reaction moment. Therefore, we have:

(10) $$2F_{SMA}\left(r+e\right)=EI\kappa$$

where $F_{SMA}$ is the force provided by a single SMA wire. Meanwhile, the strain of a single SMA wire can be expressed as:

(11) $$\left(r+e\right)\kappa =-\left(\varepsilon_{0}+\frac{F_{SMA}}{E_{A}A}\right)$$

where $E_{A}$ is the Young’s modulus of SMA at the austenite phase and *A* is the cross-section area of the SMA wires. Given that the cross-section can be replaced by the diameter of the SMA wire, $d_{0}$, by combining the aforementioned Equations (1), (8), (10) and (11), the curvature of the distal actuator can therefore be solved as:

(12) $$\kappa =-\frac{\varepsilon_{0}}{r+e+\frac{4EI}{2\pi {d_{0}}^{2}E_{A}(r+e)}}$$

### 3.2. Simulation by Finite Element Method

In this section, the mathematical model is verified using the finite element method (FEM). The distal actuator is simplified as a PE base embedded with two shape memory alloy wires, and its deformation capability is simulated using the ABAQUS finite element simulation software. The material properties required for the simulation are given in Table 1. It is important to point out that a negative coefficient of thermal expansion (CTE) is introduced to describe the thermal contraction due to the SME. The constraints between the PE base and SMA wires of the finite element model are set as tie constraints, and its boundary conditions are assumed as one end fixed and the other end free. To incorporate the temperature–displacement coupling, an appropriate thermal field with the same temperature as the austenite finish temperature of the SMA is loaded on the finite element model.

**Figure 5:**
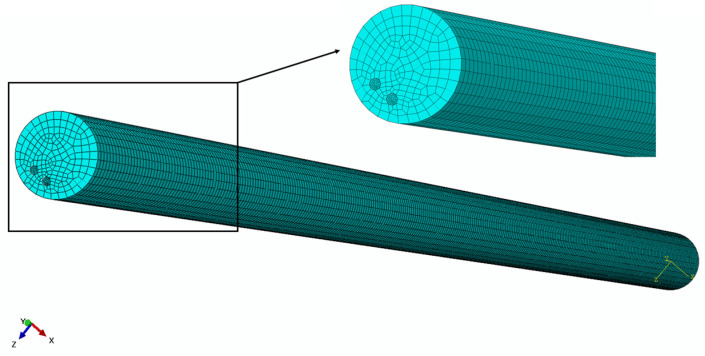
Mesh division diagram of the simulation model.

**Figure 6:**
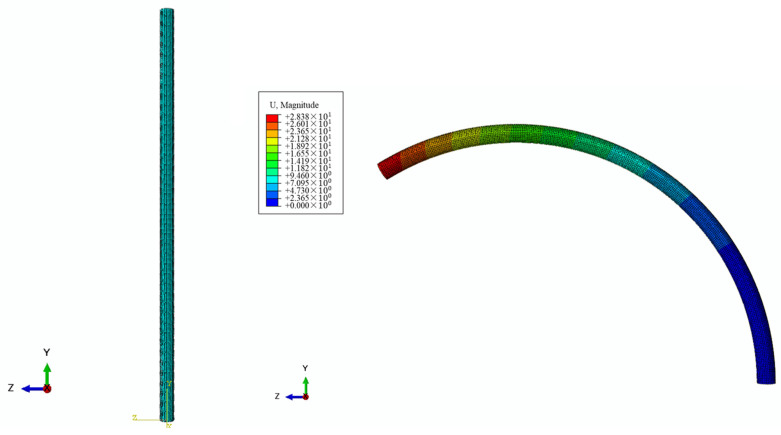
Simulation solution of a representative structure with 1 mm diameter and 0.3 mm eccentric distance.

In addition to the aforementioned settings, the mesh size also plays a crucial role in the accuracy of the simulation model. For this study, the mesh size was set at 0.1 mm for the PE base and 0.014 mm for the SMA wire, as depicted in Figure 5. Convergence analysis on the finite element result regarding meshing settings was completed, and it was proven that the mesh sizes for PE and SMA are available for finite element results with sufficient precision. Moreover, a typical finite element result, as seen in Figure 6, shows the initial state of the model and its deformed state. Based on the aforementioned specified settings and boundary conditions, the elastic PE base is driven to deform significantly by the embedded U-shaped SMA wire.

**Figure 7:**
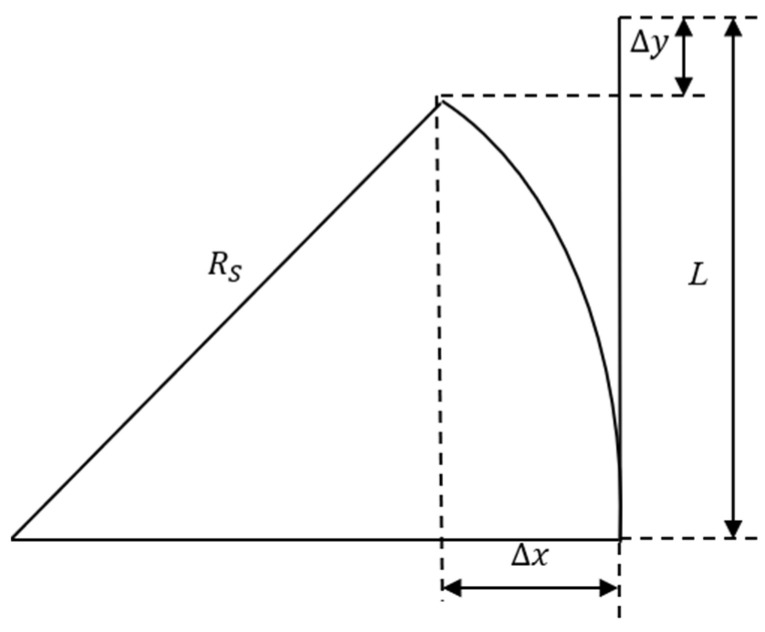
Analysis of deformation result solved by the simulation model.

To determine the curvature of the deformed finite element model of the distal actuator, the relationship between its tip displacements and its curvature is required. Based on the simulation results, it is evident that the model undergoes deflection towards a specific direction due to the contraction of the SMA wire, as depicted in Figure 7. The acquired curvatures from the finite element models can thus be compared with those obtained by the analytical model.

Clearly, the tip of the deformed distal actuator of length L can be decomposed into the displacements ∆x and ∆y along the x axis and y axis, respectively. According to the geometric relationship shown in Figure 7, the formulation between these structural parameters and deformation parameters can be written as:

(13) $${\left(R_{S}-∆x\right)}^{2}+{\left(L-∆y\right)}^{2}={R_{S}}^{2}$$

By solving Equation (13), the curvature of the finite element model of the distal actuator can be obtained as:

(14) $$\kappa =\frac{1}{R_{S}}=\frac{2∆x}{{∆x}^{2}+{∆y}^{2}+L^{2}-2∆yL}$$

Therefore, the curvature of the distal actuator can be acquired from the geometric deformation of the finite element model, and further compared with the analytical solution given by Equation (12). The influence of various structural parameters, such as the radius of the PE base *R*, the eccentric distance *e*, and the negative thermal strain $\varepsilon_{0}$, on the deformation amplitude of the distal actuator, was studied. 

**Figure 8:**
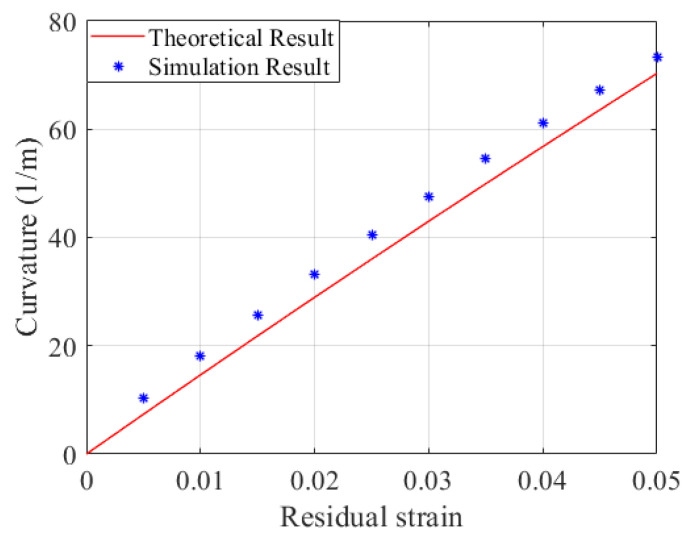
Comparison of theoretical results and simulation results for different residual strains.

Given that the tip part of the U-shaped SMA wire at the free end has little impact on the deformation of the distal actuator, the structure of the SMA wire is simplified to a PE matrix embedded with two SMA wires in the parametric study. As shown in Figure 8, when the residual strain of SMA wire increases from 0 to 5%, the curvature of the distal actuator also increases. The simulation results given by finite element models show great agreement with the theoretical solutions. The errors between the theoretical and simulation results can be partly explained by mesh and ideal constraint settings in the finite element modeling.

**Figure 9:**
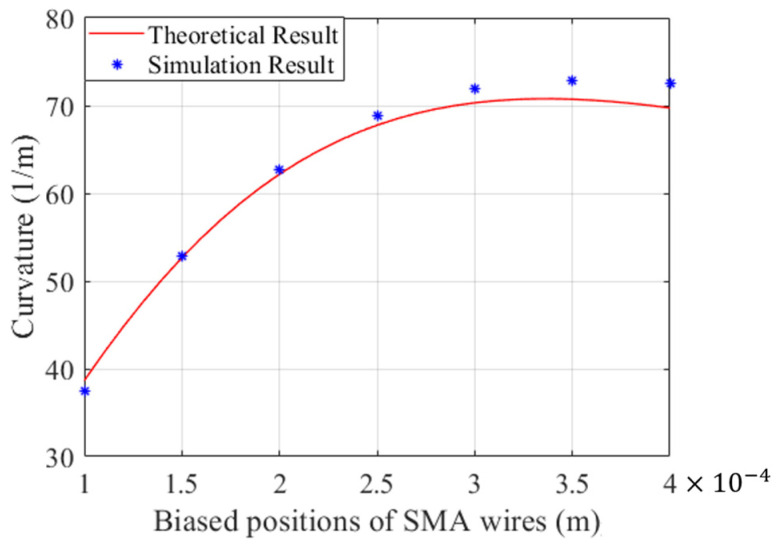
Comparison of theoretical and simulation results when the eccentricity distance of SMA wires varies.

The biased position of the SMA wire can also dramatically affect the deflection of the distal actuator. In this part, a PE base with a constant radius of 0.5 mm is employed and embedded into the PE base. Expected curvatures of the distal actuator regarding the biased positions of SMA wires were investigated and are drawn in Figure 9. The theoretical results given by the analytical model reveal that the curvature initially increases and then gradually decreases as the distance between the SMA wires and the center of the PE base increases. This trend is consistent with the simulation results obtained by finite element models, which also demonstrates the reliability of the proposed mathematical model for predicting the bending of the distal actuator. It is obvious that the maximum curvature occurs when an SMA wire with a diameter of 0.3 mm is embedded into the PE base with a diameter of 0.5 mm. Moreover, it is also worth noting that the errors between the theoretical and simulation results grow with the biased position of the SMA wire, which can be partly attributed to meshing settings.

**Figure 10:**
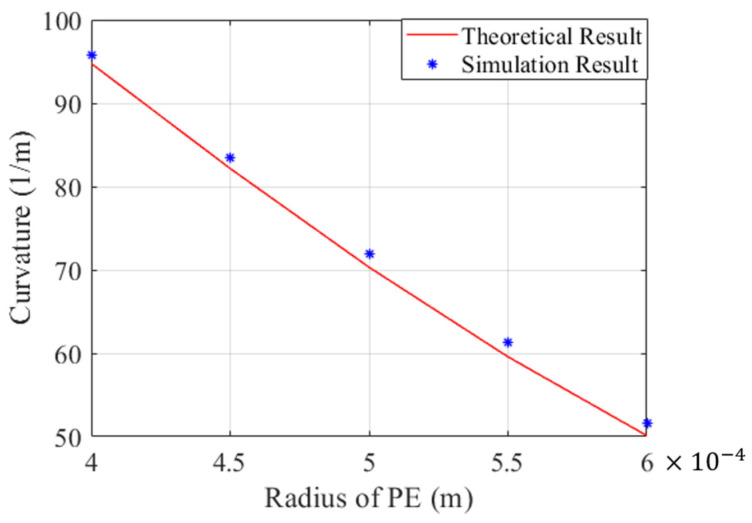
Comparison of theoretical and simulation results when the radius of PE varies.

In addition to the residual strain and biased position of the SMA wire, determination of the optimal radius of the PE base for narrow and complex vessels is also critical. In this part, a SMA wire with a diameter of 0.3 mm is selected for embedding into the PE base, and the influence of the diameter of the PE base on the curvature of the distal actuator is discussed. The theoretical results obtained by the mathematical model, as well as the simulation results acquired from the finite element mode, are drawn in Figure 10. The conclusion can be safely drawn that the curvature of the distal actuator decreases with regard to the increase in the diameter of the PE base. The high agreement between the theoretical and simulation results also verifies the accuracy of the mathematical model for rapid prediction and optimization of the distal actuator of the microcatheter.

## 4. Fabrication and Experiment

In this work, Young’s moduli of the SMA wires in martensitic and austenitic phases were characterized using a tensile testing device. These parameters are subsequently employed in the mathematical model and finite element models. The parametric study in Section 3 demonstrates the feasibility of quick estimation of the expected bending deformation through the mathematical model and its analytical solution. Based on the aforementioned analysis and simulation, in this section, we present the fabrication of a representative distal actuator of the proposed microcatheters for experimental verification. According to the proposed fabrication shown in Figure 3, SMA wires with a diameter of 0.1 mm and a linear steel mold with an inner diameter of 0.72 mm are chosen. Firstly, the SMA wire is detwinned to reserve a certain residual strain under a proper tensile force provided by the tensile testing device. Subsequently, the liquid PE is injected into the mold after the detwinned SMA wire is biasedly positioned in the steel sheath mold. After natural curing in the air, the cured PE with the biasedly embedded SMA wire can be pulled out of the steel mold. To ensure safe demolding of the distal actuator, the tensile testing device is utilized for the demolding process with in situ detection of the demolding force.

**Figure 11:**
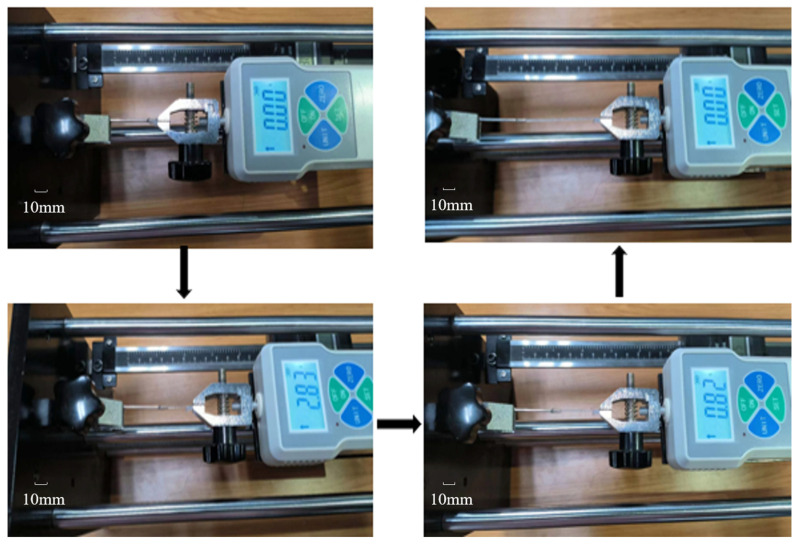
The demolding process of the distal actuator by the tensile test device.

During the demolding process, it can be observed that the tensile force applied to the distal actuator initially increases and then decreases once the pulling force exceeds the peeling force between the PE base and the steel mold, as illustrated in Figure 11. The unit of the dynamometer is Newton, and the displacement measurement device on the tensile test device is measured in millimeters. It is also worth pointing out that the loading speed should not be too fast to avoid damaging the distal actuator. In this study, a single-step displacement increment per second of the tensile test device was used to measure the tensile speed, which is recommended not to exceed 0.5 mm/s to avoid damage to the distal actuator. Once the loading speed of the tensile stress increases to beyond 0.5 mm/s, the adhesion between the SMA wire and the silicone rubber is too weak to keep the stable driving of the distal body. Therefore, it is necessary to properly control the loading speed during the machining process to ensure that the distal actuator can be reliably removed from the mold.

The prototype of the proposed microcatheter can be assembled by combining a matched PE catheter with the distal actuator, which is demolded from the steel mold during the fabrication process described above. The deformability of the distal actuator plays a key role in the development and operation of active microcatheter. In this part, an experiment was conducted to validate the deformation ability of the distal actuator by applying a constant current to the SMA wire of the distal actuator. The thermal field and deformation were detected in situ using an infrared camera (FLIR A655sc), and thus the relationship between the deflection of the distal actuator and the real-time temperature distribution across the distal actuator was obtained.

**Figure 12:**
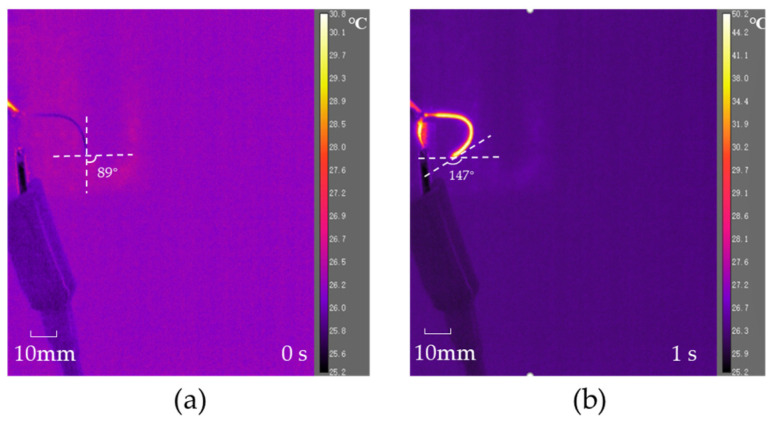
Infrared images of the distal actuator in different situations: (**a**) power-off; (**b**) power-on.

**Figure 13:**
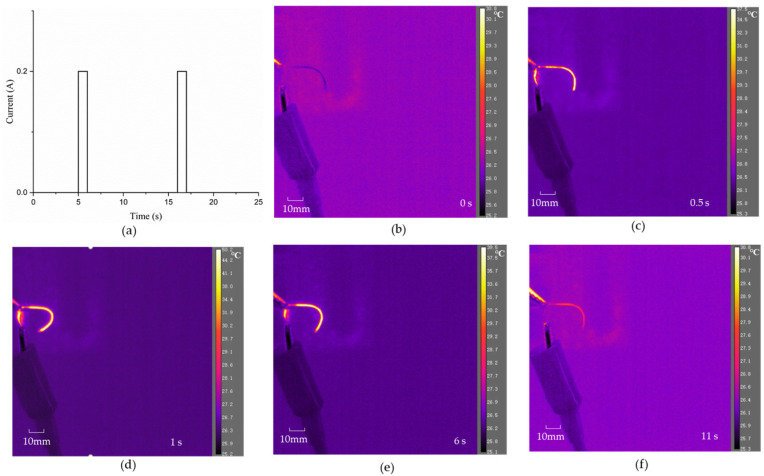
In situ electro-thermal cyclic test on the distal actuator: (**a**) current loading; (**b**–**f**) reconfiguration process during a cyclic test.

It is worth noting that the length of the distal actuator also has an important impact on the realization of its active guidance function. Due to its small design diameter and weak stiffness, it is difficult to maintain stability with a large length. Therefore, it is not recommended that the length be greater than 50 mm, which is long enough for distal actuators. Specifically, a distal actuator with a length of 38 mm and a diameter of 0.72 mm was fabricated. The SMA wire used for the distal actuator has a diameter of 0.1 mm and its phase transformation temperature is approximately 35 °C. An infrared camera was employed to characterize the deformation process of the designed distal actuator under Joule heating. In the initial state, it can be seen from Figure 12a that the initial configuration of the distal actuator is not a linear shape, which can be explained by the non-ideal physical bonding between the SMA wire and the PE base. Significant reconfiguration of the distal actuator is achieved when a constant current power is applied to the SMA wire embedded in the PE base, as shown in Figure 12b. A bending angle change from about 89° to nearly 147° is detected. The applied current for this electro-thermally cyclic loading is seen in Figure 13a, which plots two full cycles of current loading. In each cycle, a 0.2 A current was applied to the 0.1 mm SMA wire for 1 s, followed by naturally cooling in air for 10 s. Figure 13b–e show representative configurations of such a distal actuator during the in situ electro-thermal loading test. The in situ electro-thermal testing on active reconfiguration of the distal actuator is recorded in the supplementary video, indicating effective control of such distal actuators for efficient navigation in narrow and complex surroundings including vessels. It is worth noting that the applied current can be adjusted according to the diameter of the SMA wire to ensure that the balanced temperature of the SMA, when driven by Joule heating, is higher than its austenite finish temperature.

## 5. Discussion and Conclusions

The focus of this paper primarily lies in investigating the effects of the size of PE, residual strain, and eccentricity distance of the SMA wires on the curvature of the distal actuator. These findings are important for optimizing the overall structure of the microcatheter. The distal actuator for active microcatheters is of significant importance in interventional surgery, as it possesses miniaturization capabilities and an active guidance function that can greatly reduce the workload on doctors. However, the current research is not exhaustive, and further work is required to facilitate its clinical application. This paper only presents preliminary qualitative experiments, and it is imperative to optimize the processing technology to perform quantitative experiments that can be compared with simulation results, thereby verifying the accuracy of the mathematical model. Moreover, simulations and tests in a blood environment are necessary due to the complex nature of the microcatheter. Additionally, incorporating environmental sensing functions into the design of microcatheters should be considered.

In summary, a novel distal actuator for active microcatheter is proposed, and was fabricated and experimentally demonstrated, in this work. By biasedly embedding a U-shaped SMA wire into the submillimeter PE elastic base, the assembled distal actuator can be effectively and efficiently driven by the shape memory effect. A mathematical model is proposed and proven reliable for rapid prediction of the bending curvature of the distal actuator. Parametric studies on such critical parameters as the diameter and the biased position of the SMA wire and the diameter of the PE base reveal that the theoretical results given by the mathematical model are in good agreement with the simulation results obtained by the finite element model. The proposed fabrication process of the distal actuator was experimentally proven to be feasible, and a typical prototype of this distal actuator, consisting of a U-shaped SMA wire with a diameter of 0.1 mm and a 38 mm long PE base with a diameter of 0.72 mm, was successfully developed. Reconfiguration of the fabricated distal actuator was realized by a direct-current power supply that enables balanced temperature heating above the austenite finish temperature of the SMA-embedded wires. Significant thermo-mechanical deformation of the distal actuator was detected in situ, which indicates the promising applications of the active modular as a microcatheter.

## Figures and Tables

**Table 1:** Properties of materials used in ABAQUS.

Material	Density (10^−9^ t/mm^3^)	Young’s Modulus (MPa)	CTE (1/°C)
SMA	6.45	66,000	−0.0005
PE	0.95	1070	0.0002

## Data Availability

The original contributions presented in the study are included in the article, further inquiries can be directed to the corresponding authors.

## Supplementary Materials

The following supporting information can be downloaded at: https://www.mdpi.com/article/10.3390/mi15050603/s1. Supplementary video: In-situ characterization on active reconfiguration of an electro-thermally driven distal actuator.
